# Supplementary material for: Identification of levoglucosan degradation pathways in bacteria and sequence similarity network analysis
Source: Arch Microbiol. 2023 Mar 31;205(4):155. doi: 10.1007/s00203-023-03506-y (PMC10066097; doi:10.1007/s00203-023-03506-y)
Supplement: Supplementary file 1 — Supplementary file1 (DOCX 6072 KB) [file 203_2023_3506_MOESM1_ESM.docx]

**SUPPLEMENTARY INFORMATION**

**Identification of levoglucosan degradation pathways and genomic analysis in bacteria**

Arashdeep Kaur,^1,2^ Nichollas Scott,^3^ Marion Herisse,^3^ Ethan D. Goddard-Borger,^4,5^ Sacha Pidot,^3^ Spencer J. Williams*^1,2^

^1^ School of Chemistry, University of Melbourne, Parkville, Victoria 3010, Australia.

^2^ Bio21 Molecular Science and Biotechnology Institute, University of Melbourne, Parkville, Victoria 3010, Australia

^3^ Department of Microbiology and Immunology, University of Melbourne, at the Peter Doherty Institute for Infection and Immunity, Victoria, 3000, Australia

^4^ Department of Medical Biology, University of Melbourne, Parkville, Victoria 3010, Australia

^5^ ACRF Chemical Biology Division, The Walter and Eliza Hall Institute of Medical Research, Parkville, Victoria 3010, Australia

E-mail: [sjwill@unimelb.edu.au](mailto:sjwill@unimelb.edu.au); ORCID: 0000-0001-6341-4364

import pandas as pd

import sqlite3

con=sqlite3.connect("Data/LGDH_neighbors_output.sqlite")

df = pd.read_sql_query("SELECT accession from neighbors", con)

con.close()

df.to_csv('Data/LGDH_neighbors.list', header=False, index=False)

**Figure S1.** Python script used to extract the accession codes of the retrieved neighbors from the SQlite file downloaded from EFI-GNT tools.

**
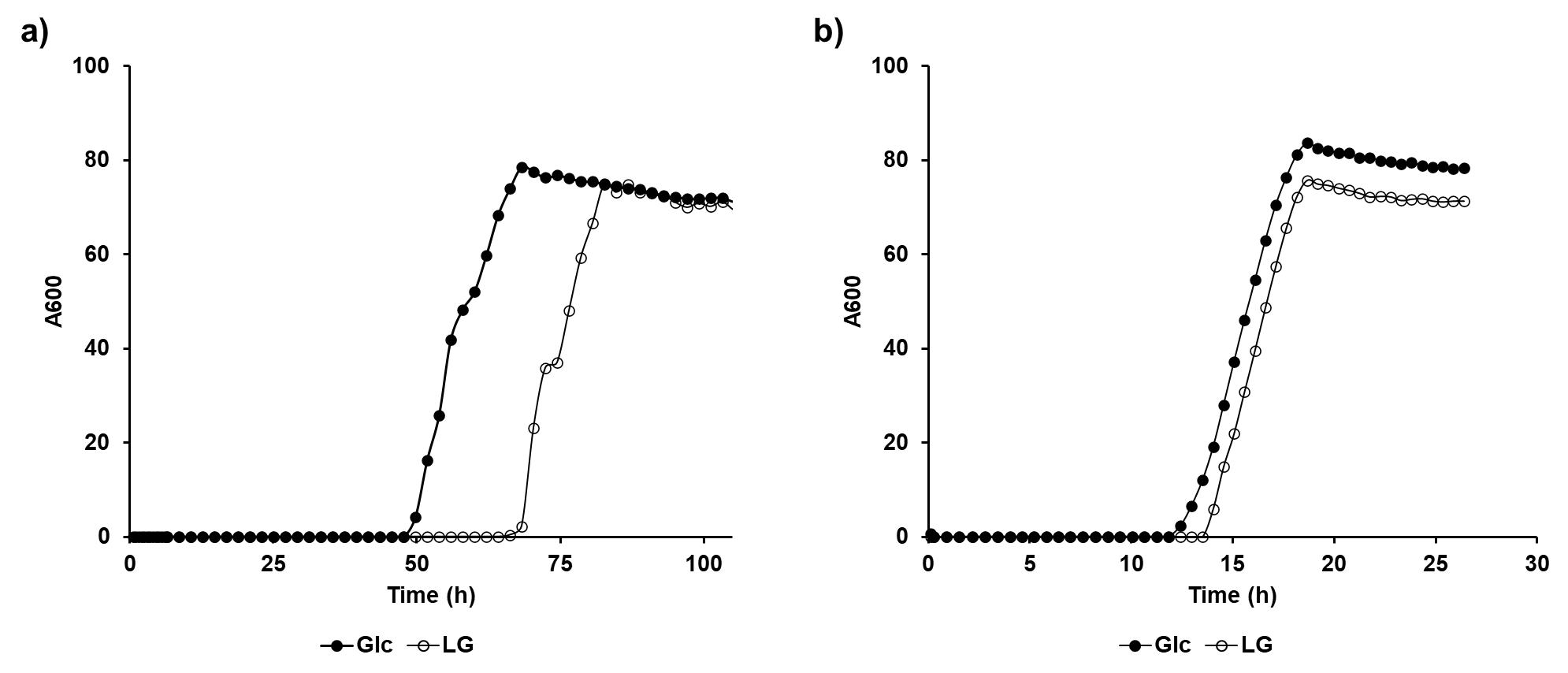
**

**Figure S2.** Growth curves of *Paenarthrobacter* strains a) LG01 and b) LG02 grown on M9 media containing 5 mM glucose or SQ.


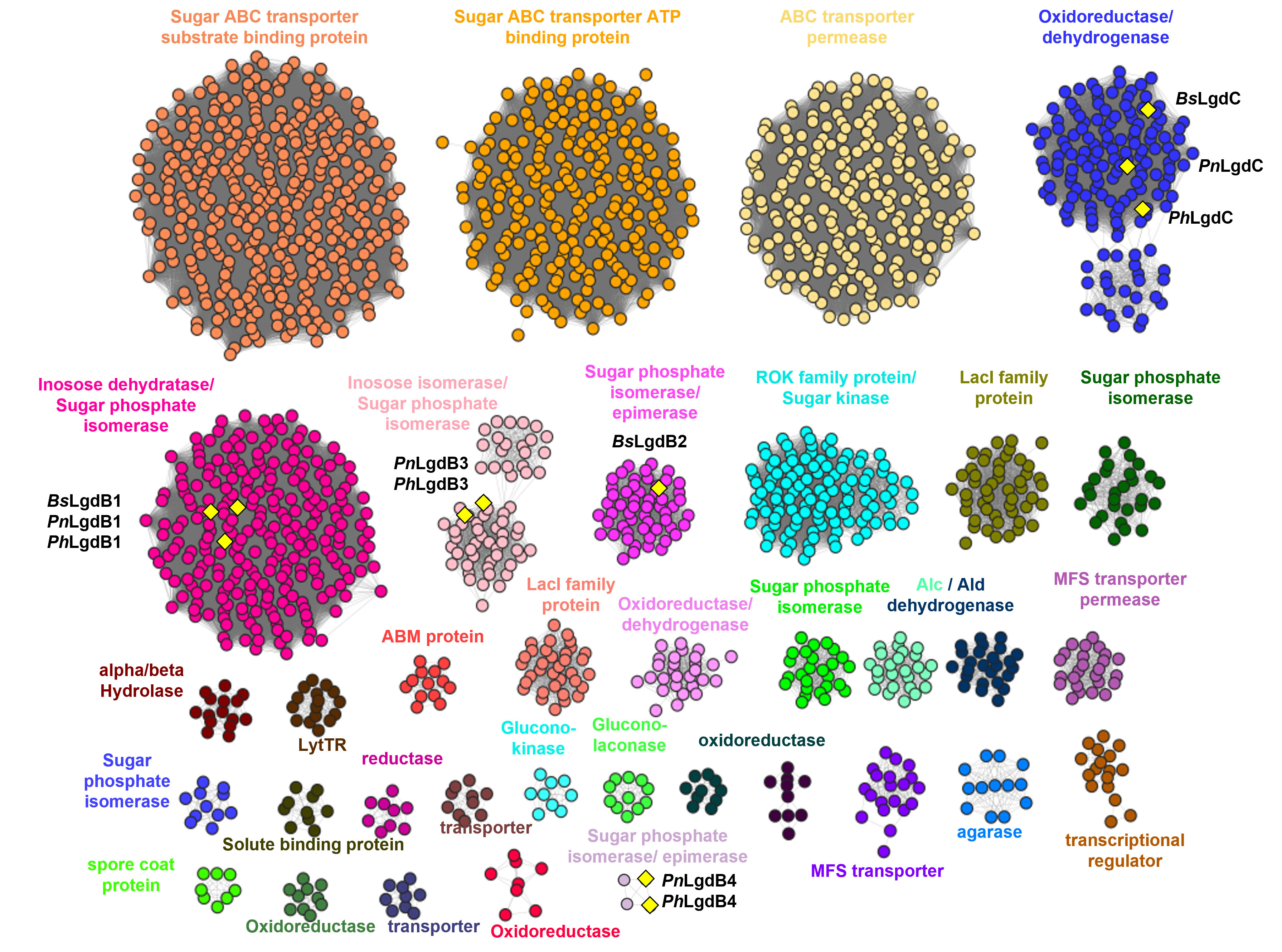


**Figure S3.** Sequence similarity network of neighbours (SSNN) of LgdA. SSNN was generated with an alignment score of 50. Isofunctional clusters are coloured accordingly to their assigned function. Three large clusters are for ABC transporters (orange, yellow and light yellow). LgdB1 and LgdB2 map onto two clusters coloured bright pink and magenta. LgdC maps onto a blue cluster. Additional clusters annotated as inosose dehydratase/sugar phosphate isomerase (light pink) and sugar phosphate isomerase/epimerase (new one) contain proteins annotated in this work as LgdB3 and LgdB4. Yellow diamonds denote proteins that have been studied previously or in this study. From *Bacillus smithii* S-2701M: *Bs*LgdB1, *Bs*LgdB2, *Bs*LgdC; from *Paenarthrobacter nitrojuajacolis* LG01: *Pn*LgdB1, *Pn*LgdB3, *Pn*LgdC; from *Paenarthrobacter histidinolovorans* LG02: *Ph*LgdB1, *Ph*LgdB3, *Ph*LgdC. Clusters smaller than 7 members are not shown, except for the cluster for LgdB4.

**Table S1. Classification and general feature of *Paenarthrobacter* spp. LG01 and LG02**

| **MIGS ID** | **Property** | **Term** | **Evidence code** |
| --- | --- | --- | --- |
|  | Classification | Domain *Bacteria* | TAS [1] |
|  |  | Phylum *Actinobacteria* | TAS [2] |
|  |  | Class *Actinomycetia* | TAS [3] |
|  |  | Order *Micrococcales* | TAS [3-6] |
|  |  | Family *Micrococcaceae* | TAS [3-5, 7] |
|  |  | Genus *Paenarthrobacter* | TAS [8] |
|  |  | Species *Paenarthrobacter nitrojuajacolis* LG01 | TAS [8] |
|  |  | Species *Paenarthrobacter histidinolovorans* LG02 | IDA |
|  | Gram Strain | Not measured |  |
|  | Cell Shape | Not reported |  |
|  | Motility | Not reported |  |
|  | Sporulation | Not reported |  |
|  | Optimum Temperature | Not tested, used 30℃ | IDA |
|  | pH range; Optimum | Not tested, used 7-8 | IDA |
|  | Carbon source | Yeast extract/tryptone, glucose, levoglucosan | IDA |
| MIGS-6 | Habitat | Soil | IDA |
| MIGS-22 | Oxygen requirement | Aerobic | IDA |
| MIGS-15 | Biotic relationship | Free living | IDA |
| MIGS-4 | Geographic location | Melbourne, VIC, Australia | IDA |
| MIGS-5 | Sample collection | June 11, 2021 | IDA |
| MIGS-4.1 | Latitude | -37.7949428 | IDA |
| MIGS-4.2 | Longitude | 144.9525146 | IDA |
| MIGS-4.4 | Altitude | Not reported |  |

*Evidence codes - IDA: Inferred from Direct Assay; TAS: Traceable Author Statement (i.e., a direct report exists in the literature)

**Table S2. Table of accession codes for levoglucosan degrading genes.**

| **Annotation** | **Description** | **Accession code** |
| --- | --- | --- |
| ***Bs*LgdA** | levoglucosan dehydrogenase | BCB28822.1 |
| ***Bs*LgdB1** | β-eliminase | BCB28820.1 |
| ***Bs*LgdB2** | 3-keto-glucose-dehydratase | BCB28827.1 |
| ***Bs*LgdC** | glucose-3-dehydrogenase | BCB28826.1 |
| ***Pp*LgdA** | levoglucosan dehydrogenase | ADX72256.1 |

*Bs* = *Bacillus smithii* S-2701M, *Pp* = *Pseudarthobacter phenanthrenivorans* Sphe3.

**Table S3. Percentage identity for LG degrading proteins: Oxidoreductases LgdA and LgdC.**

|  | ***Pp*LgdA** | ***Bs*LgdA** | ***Pn*LgdA** | ***Ph*LgdA** | ***Pn*LgdC** | ***Ph*LgdC** |
| --- | --- | --- | --- | --- | --- | --- |
| ***Pp*LgdA** |  | **70.1** | **96.7** | **97.9** | 33.3 | 33.3 |
| ***Bs*LgdA** |  |  | **71.5** | **70.7** | 32.2 | 31.9 |
| ***Pn*LgdA** |  |  |  | **97.4** | 32.3 | 32.9 |
| ***Ph*LgdA** |  |  |  |  | 32.9 | 32.9 |
| ***Pn*LgdC** |  |  |  |  |  | **96.9** |
| ***Ph*LgdC** |  |  |  |  |  |  |

Bold shows percentage identity >50%. *Pp* = *Pseudarthrobacter phenanthrenivorans* Sphe3, *Bs* = *Bacillus smithii* S-2701M, *Pn* = *Paenarthrobacter nitrojuajacolis* LG01 and *Ph* = *Paenarthrobacter histidinolovorans*

**Table S4. Percentage identity for LG degrading proteins: LgdB1, LgdB2, LgdB3, LgdB4 (annotated as sugar phosphate isomerase).**

|  | ***Bs*LgdB1** | ***Bs*LgdB2** | ***Pn*LgdB1** | ***Pn*LgdB3** | ***Pn*LgdB4** | ***Ph*LgdB1** | ***Ph*LgdB3** | ***Ph*LgdB4** |
| --- | --- | --- | --- | --- | --- | --- | --- | --- |
| ***Bs*LgdB1** |  | 31.7 | **55.5** | 22.0 | 27.6 | **55.6** | 25.4 | 30.5 |
| ***Bs*LgdB2** |  |  | 24 | 26.7 | - | 23.0 | 26.4 | **-** |
| ***Pn*LgdB1** |  |  |  | - | 25.6 | **97.4** | 23.4 | 25.2 |
| ***Pn*LgdB3** |  |  |  |  | 23.2 | 26.1 | **97.6** | 24.0 |
| ***Pn*LgdB4** |  |  |  |  |  | 29.9 | 22.8 | **91.5** |
| ***Ph*LgdB1** |  |  |  |  |  |  | 22.7 | 29.9 |
| ***Ph*LgdB3** |  |  |  |  |  |  |  | - |
| ***Ph*LgdB4** |  |  |  |  |  |  |  |  |

Bold shows percentage identity >50%. *Pp* = *Pseudarthrobacter phenanthrenivorans* Sphe3, *Bs* = *Bacillus smithii* S-2701M, *Pn* = *Paenarthrobacter nitrojuajacolis* LG01 and *Ph* = *Paenarthrobacter histidinolovorans.*

**Table S5. RMSD values for structural comparison: Oxidoreductases LgdA and LgdC.**

|  | ***Pp*LgdA** | ***Pn*LgdA** | ***Bs*LgdC** | ***Pn*LgdC** |
| --- | --- | --- | --- | --- |
| ***Pp*LgdA** |  | 0.252 | 0.869 | 0.963 |
| ***Pn*LgdA** |  |  | 0.882 | 0.994 |
| ***Bs*LgdC** |  |  |  | 0.641 |
| ***Pn*LgdC** |  |  |  |  |

**Table S6. RMSD values for structural comparison: LgdB1, LgdB2, LgdB3, LgdB4 (annotated as sugar phosphate isomerase).**

|  | ***Bs*LgdB1** | ***Bs*LgdB2** | ***Pn*LgdB1** | ***Pn*LgdB3** | ***Pn*LgdB4** |
| --- | --- | --- | --- | --- | --- |
| ***Bs*LgdB1** |  | 2.705 | 0.433 | 5.326 | 2.218 |
| ***Bs*LgdB2** |  |  | 2.782 | 1.345 | 3.288 |
| ***Pn*LgdB1** |  |  |  | 11.361 | 1.443 |
| ***Pn*LgdB3** |  |  |  |  | 7.613 |
| ***Pn*LgdB4** |  |  |  |  |  |

**References**

1. Woese CR, Kandler O, Wheelis ML. Towards a natural system of organisms: proposal for the domains Archaea, Bacteria, and Eucarya. Proc Natl Acad Sci USA. 1990;87: 4576.

2. Ludwig W, Euzéby J, Schumann P, Busse H-J, Trujillo ME, Kämpfer P, et al. Road map of the phylum Actinobacteria. In: Goodfellow M, Kämpfer P, Busse H-J, Trujillo ME, Suzuki K-i, Ludwig W, et al., editors. Bergey’s Manual® of Systematic Bacteriology: Volume Five The Actinobacteria, Part A and B. New York, NY: Springer New York; 2012. p. 1-28.

3. Stackebrandt E, Rainey FA, Ward-Rainey NL. Proposal for a New Hierarchic Classification System, *Actinobacteria classis* nov. 1997;47: 479-91.

4. Zhi X-Y, Li W-J, Stackebrandt E. An update of the structure and 16S rRNA gene sequence-based definition of higher ranks of the class Actinobacteria, with the proposal of two new suborders and four new families and emended descriptions of the existing higher taxa. Int J Syst Evolut Microbiol. 2009;59: 589-608.

5. Skerman VBD, McGOWAN V, SNEATH PHA. Approved Lists of Bacterial Names. Int J Syst Evolut Microbiol. 1980;30: 225-420.

6. Buchanan RE. Studies in the nomenclature and classification of the bacteria II. The primary subdivisions of the Schizomycetes. J Bacteriol. 1917;2: 155-64.

7. Pribram E. A contribuiton to the classification of the microorganisms. J Bacteriol. 1929;18: 361-94.

8. Busse HJ. Review of the taxonomy of the genus *Arthrobacter*, emendation of the genus *Arthrobacter sensu lato*, proposal to reclassify selected species of the genus *Arthrobacter* in the novel genera *Glutamicibacter* gen. nov., *Paeniglutamicibacter* gen. nov., *Pseudoglutamicibacter* gen. nov., *Paenarthrobacter* gen. nov. and *Pseudarthrobacter* gen. nov., and emended description of *Arthrobacter roseus*. Int J Syst Evol Microbiol. 2016;66: 9-37.
